# Is anybody doing it? An experimental study of the effect of normative messages on intention to do physical activity

**DOI:** 10.1186/1471-2458-14-778

**Published:** 2014-07-31

**Authors:** René van Bavel, Gabriele Esposito, Tom Baranowski

**Affiliations:** Institute for Prospective Technological Studies, Joint Research Centre, European Commission, Calle Inca Garcilaso 3, 41092 Seville, Spain; Children’s Nutrition Research Center, Department of Pediatrics, Baylor College of Medicine, Houston, TX USA

**Keywords:** Physical activity, Normative messages, Health behaviour, Young adults

## Abstract

**Background:**

The study explores whether messages about the physical activity levels of the majority (i.e. normative messages) affect young adults' intention to engage in regular physical activity.

**Methods:**

An experimental survey among 16 to 24 year-olds in Bulgaria, Croatia and Romania (n = 1200) was conducted in March 2013. A control group received no message; one treatment group was told that the majority was physically active (positive message); and another treatment group was told that the majority was not physically active (negative message).

**Results:**

Both the positive and (unexpectedly) the negative normative messages showed a significant and positive effect on intention to be physically active. There was no difference between the effects of the messages.

**Conclusions:**

Normative messages affect intention, which is encouraging for public health campaigns. The effect of the positive message confirms previous findings on conformity to the norm; the effect of the negative message is unexpected and requires further research to be understood.

## Background

Participating in regular physical activity has significant health benefits. It can improve health and quality of life by strengthening the cardiovascular system, reducing the risk of type 2 diabetes, maintaining a degree of psychological well-being, and controlling body weight [1, 2]. A number of public health institutions recommend approximately 30 minutes of moderate to vigorous physical activity on most days of the week [3–5]. A large part of the population in western countries appears to be aware of these benefits [6], but still fails to meet these recommendations, continuing to live a sedentary lifestyle [7]. Young people (16–24 years old) are notoriously difficult to reach through public health campaigns [8] and are particularly vulnerable to weight gain [9, 10].

Public health campaigns have sought to increase people's physical activity. Their relative lack of success in changing behaviour suggests there is room for improvement [11, 12]. Psychological insights may improve public health interventions in general, and physical activity campaigns in particular [13]. For example, the effectiveness of public health messages on physical activity may depend on the benefits being framed in terms of health gains or losses [14]. A targeted approach of this kind could be particularly beneficial for promoting increased levels of physical activity among 16–25 year olds [8].

The role of social norms in promoting physical activity merits further investigation. Evidence suggests that *injunctive norms* (i.e. perceptions of what other people think one should do) are not particularly effective in promoting physical activity for this crucial age group [11]. On the other hand, *descriptive norms* (i.e. perceptions of what most other people are doing, sometimes referred to as 'social proof' [15]) may hold greater promise, since the behaviour of others is often taken as a cue to what should be done [16].

The persuasive impact of descriptive norms has already been studied and applied to fields other than physical activity. In appeals for hotel guests to reuse their towels, for example, messages using descriptive norms were more effective than traditional messages [17]. Moreover, these normative messages capitalized on the 'provincial effect': the more proximal the group, the more influential its behaviour. Messages were most effective when describing the behaviour of guests who had stayed *in the same room*, as opposed to the behaviour of hotel guests in general. This particular insight was also applied in the UK to get people to pay their taxes on time [18].

Evidence of the impact of descriptive norms on physical activity is mixed. In some cases, it has not been demonstrated [19]. Other studies, however, have shown an association between peer norms and adolescents' attitudes and intentions to engage in physical activity [20, 21]. One study established – precisely – that exposure to messages containing descriptive norm information about the prevalence of others' physical activity positively affected individual physical activity behaviour. However, a second study attempting to extend these results found no effect [22]. Evidence also supports the existence of a provincial effect in physical activity. Friends’ physical activity was more strongly related to individual activity than the physical activity of other groups [23], and descriptive norms associated with friends' physical activity were the strongest predictors of behaviour [24].

The present study introduced behavioural insights into public health messages on physical activity [11] and used targeted messages building on the provincial effect [17]. The objective was to test whether exposure to normative messages affected people's intention to be physically active. Subjects were exposed to either positive or negative messages (suggesting that the majority of people did, or did not, do physical activity) and were later asked about their intention to be physically active. The guiding hypotheses, following the established knowledge on conformity to the norm, were that (a) subjects exposed to the positive message would indicate a greater intention to engage in physical activity than those not exposed to a normative message; and (b) subjects exposed to the negative message would indicate a lesser intention to engage in physical activity than those not exposed to a normative message.

The study was conducted in three countries, Bulgaria, Croatia and Romania, to increase the generalizability of results. South-eastern Europe was chosen because the economic and political transition following the end of Communist regimes and the break-up of Yugoslavia left the region with weakened public health infrastructures, making it a propitious setting for a study with public health implications [25]. In addition, these countries are relevant from a public health perspective because they are the least convinced in Europe of the effectiveness of physical activity promotion campaigns to reduce the spread of obesity among children. Only 5% of the population in Bulgaria, 7% in Croatia and 10% in Romania are convinced, compared to the European average of 14% [26].

## Methods

To study the effect of messages about the prevalence of physical activity on intention to engage in physical activity, an experimental on-line survey was conducted in collaboration with Block de Ideas, a social research company based in Barcelona. The internal Evaluation Committee set up at the Institute for Prospective Technological Studies approached the study as a questionnaire with a split-ballot and sought adherence to the appropriate ethical guidelines for conducting surveys. Informed consent was obtained from all participants to the study following the guidelines of ESOMAR, the World Association for Social, Opinion and Market Research (including their Guideline on Interviewing Children and Young People)^1^. Their data were anonymised, kept confidential and used only for the purpose of this research.

The on-line survey had a total a sample of 1200 young Internet users across all three countries, aged 16–24, as part of a European Commission study on physical activity in selected European countries. These Internet users are a good approximation to the overall population of 16–24 year-olds, as Internet access is widespread in these countries. In 2012, 83% of Bulgarians, 98% of Croatians and 77% of Romanians in this age category used the Internet in the last 3 months [27]. The size of the sample allowed for an interpretation of the results with a confidence level of 95.5% (Z = 1.96; p = q = 50) and a sampling error of +/−0.85% for the overall data and +/−3.16% for the country-specific data.

On-line questionnaire responses have been less susceptible to social desirability biases than face-to-face interviews [28]. To mitigate the risks associated with on-line surveys such as the uncertain identity of respondents or a large drop-out rate, participants were recruited from pre-existing pools which complied with a series of quality control measures. These measures included: respondents rating their activity; automatic deletion if e-mail addresses were not functioning; re-invitations to remind people to participate in the survey; detection and removal of duplicate respondents; username and password logins; and round-the-clock monitoring of servers and networks. Participants were selected at random from the pool, but the sample was designed to match the exact percentage of women, men, 16–18 year-olds and 19–24 year-olds in each country (as indicated by Eurostat) to ensure representativeness of the target population. The drop-out rate was 24%, i.e. 382 subjects decided to close the survey before the end and had to be replaced to reach the target of 1200 complete responses.

Since it was part of a larger study looking at physical activity, the questionnaire included questions on socio-demographics, well-being and health background characteristics, and past behaviour measured with the short version of the international physical activity questionnaire (IPAQ). It also included items pertaining to the theory of planned behaviour (TPB) and the model of goal-directed behaviour (MGDB). In addition, respondents were asked about their use of information and communications technology (ICT) and different information sources about physical activity and health. This article focuses on the impact of normative messages on intention, while other manuscripts describe the results of the psychometric analyses and the potentially mediating role of MGDB constructs.

The IPAQ was administered at the beginning of the survey. The final scores included a weighted average of walking, moderate activity and vigorous activity. These final scores were truncated: values greater than 180 minutes per day in any of the categories were re-coded to 180 (this happened in 268 cases for walking, 477 for moderate activity and 508 for vigorous activity); values lower than 10 minutes per day in each of these categories were recoded to 0 (this happened in one case for walking, in four for moderate activity and in none for vigorous activity).

The questionnaire was translated from English into Bulgarian, Croatian and Romanian, with a 'back-translation' quality check. The pilot phase of the survey was launched in February 2013 and, after further refinement, the survey was launched in March 2013. 106 participants took part in the pilot: 36 from Bulgaria, 33 from Croatia and 37 from Romania. As a result of the pilot, the questionnaire was shortened and pictures were added to the normative messages as described below.

Respondents were randomly assigned to one of three different groups:

- Control Group (n = 400, equally split among the three countries), where intention to engage in physical activity was tested without participants being exposed to social-norm type messages;
- Treatment 1 (n = 400, equally split among the three countries), where intention to engage in physical activity was tested after participants were exposed to a personalized message suggesting that over 79% of their peers (i.e. people of the same country, gender and age) did more than 30 minutes of moderate physical activity on most days of the week (positive message); and
- Treatment 2 (n = 400, equally split among the three countries), where this same intention was tested after participants were exposed to a personalized message suggesting that over 79% of their peers did not do more than 30 minutes of moderate physical activity on most days of the week (negative message).

**Figure 1:**
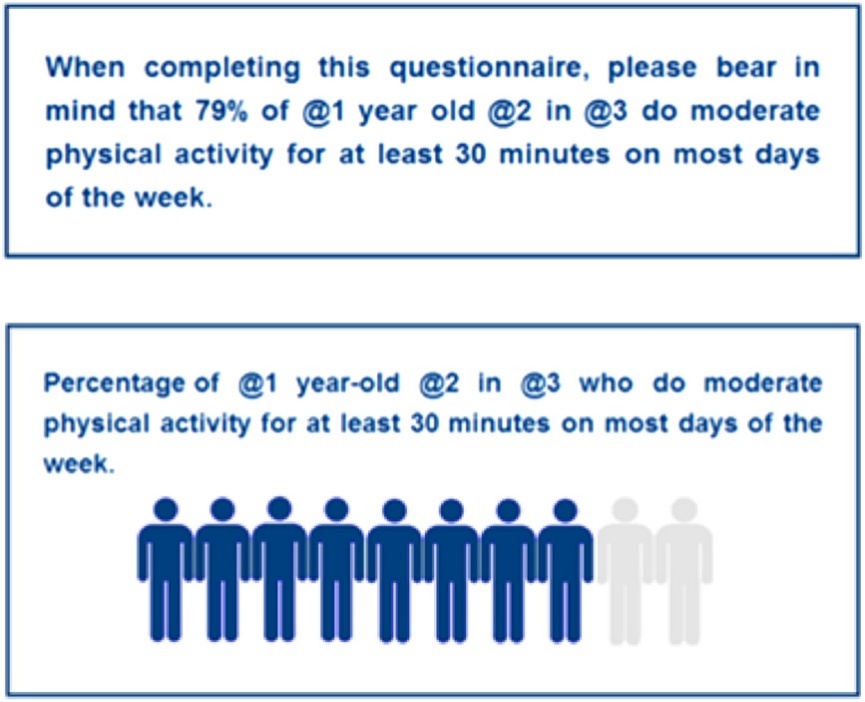
**Two screens of the positive normative message.** Messages were tailored to individual respondents: "@1" was replaced with their age, "@2" with their gender, and "@3" with their country of residence.

The positive message included three screens in succession: (a) a screen giving the message that "over 79% of [respondent's age] year-old [respondent's gender] in [respondent's country of residence] do moderate physical activity for at least 30 minutes on most days"; (b) a screen with a large picture of a young, physically attractive couple running with "79%" written alongside it together with a smaller picture of a young, physically attractive couple relaxing on a sofa with "21%" written alongside it; and (c) a screen with a graphic illustration of the percentage mentioned in the previous slide (see Figure 1). The second screen was also present, as a thumbnail, in the top right-hand corner of the screen for the remainder of the questionnaire, to reinforce the message and remind the participants of this message while they responded to each question. Subjects were exposed to each slide for 10 seconds, after which they had to click a box which said 'OK, I understand' to move to the next screen.

The negative message was designed to be the inverted equivalent of the positive message. Therefore, the message stated "over 79% of [respondent's age] year-old [respondent's gender] in [respondent's country of residence] do not do moderate physical activity for at least 30 minutes on most days". The size of the pictures was inverted, with the picture of the couple relaxing on the sofa being much larger than the picture of the couple running, and the graphic illustration also reflected these percentages.

### Measuring intention to engage in physical activity

Since physical activity was measured as part of the survey, participants did not have time to change their behaviour. Following up on respondents to observe their behaviour post treatment [11] was not feasible within the scope of this study. Therefore, the study was limited to measuring *intention to engage in physical activity*. This construct was based on previous work [29] and was composed of three statements evaluated on 7-point binary adjective response categories ranging from *yes, definitely* to *no, definitely.* These statements were *I plan to do…, I expect I will do…, I intend to do…*, each followed by *…physical activities at least 30 minutes on most days next week,* and appeared sequentially in the survey*.* The scale presented a Cronbach's alpha of 0.95.

The approach to the analysis was the same for both hypotheses. Univariate and multivariate analyses of variance (ANOVA) were conducted to look for differences in intention between treatment groups and gender, age, country and past physical activity behaviour. Scheffe’s test and Tukey’s honestly significant difference (HSD) test were used to check where these differences existed.

## Results

Table 1 shows the breakdown of the sample of the study by country, gender and age. Each of the 12 combinations of country, gender and age was calibrated to be proportional to the actual population as indicated by Eurostat. Table 1 also shows the same breakdown for the 382 participants who dropped out of the survey before completing it and were replaced to reach 1200 completed surveys. There was a difference in drop-out rates according to gender: in total, men dropped out 21.8% of the time and women 26.4%. Table 2 shows the breakdown of participants by treatment, country and gender. The allocations between treatments groups and control group are not significantly different.

**Table 1:** **Sample of the study (final participants and respondents dropped out before completion and replaced)**

Country	Gender	16-18 years old		19-24 years old		Total	
		Participated (share of total)	Dropped out (rate)	Participated (share of total)	Dropped out (rate)	Participated (share of total)	Dropped out (rate)
**Bulgaria**	Men	58 (51.3%)	21 (26.6%)	147 (51.2%)	45 (23.4%)	205 (51.3%)	66 (24.4%)
	Women	55 (48.7%)	23 (29.5%)	140 (48.8%)	56 (28.6%)	195 (48.7%)	79 (28.8%)
**Croatia**	Men	64 (51.2%)	9 (12.3%)	140 (50.9%)	34 (19.5%)	204 (51.0%)	43 (17.4%)
	Women	61 (48.8%)	23 (27.3%)	135 (49.1%)	49 (26.6%)	196 (49.0%)	72 (26.9%)
**Romania**	Men	55 (50.9%)	29 (34.5%)	149 (51.0%)	33 (18.1%)	204 (51.0%)	62 (23.3%)
	Women	53 (49.1%)	30 (36.1%)	143 (49.0%)	30 (17.3%)	196 (49.0%)	60 (23.4%)
**Total**	Men	177 (51.2%)	59 (25.0%)	436 (51.1%)	112 (20.4%)	**613 (51.1%)**	**171 (21.8%)**
	Women	169 (48.8%)	76 (31.0%)	418 (48.9%)	135 (24.4%)	**587 (48.9%)**	**211 (26.4%)**

**Table 2:** **Number of participants in the study by country, gender and treatment**

Bulgaria					
Men			Women		
Treatment 1	Treatment 2	Control	Treatment 1	Treatment 2	Control
70 (34%)	68 (33%)	67 (33%)	63 (32%)	65 (33%)	67 (34%)
**Croatia**					
**Men**			**Women**		
Treatment 1	Treatment 2	Control	Treatment 1	Treatment 2	Control
58 (28%)	71 (35%)	75 (37%)	75 (38%)	63 (32%)	58 (30%)
**Romania**					
**Men**			**Women**		
Treatment 1	Treatment 2	Control	Treatment 1	Treatment 2	Control
73 (36%)	65 (32%)	66 (32%)	61 (31%)	68 (35%)	67 (34%)

Since the questionnaire asked respondents a large number of questions related to physical activity, a confirmatory factor analysis was conducted. The intention to behave items had factor loadings all higher than 0.86 but were not normally distributed across groups.

A first univariate ANOVA was performed on the intention variable to preliminarily compare means between the treatment groups and the control group (see Table 3). The low p-value (<0.001) of the between-groups variation means that the difference between the groups are statistically significant. Results of Scheffe’s tests show that intention in the Control Group has a lower mean than in both the Positive Message Group (−0.18; p = 0.029, respectively) and the Negative Message Group (−0.25; p = 0.001). The difference between the means of intention in the Positive Message Group and the Negative Message Group (0.07; p = 0.630) are not statistically significant.

**Table 3:** **Univariate ANOVA model for intention to do physical activity**

Source	SS	Df	F	Prob > F
Between groups***	12.991	2	7.06	0.0009
**Within groups**	1101.343	1197		
**Total**	1114.334	1199		

Significance level: *** = 0.001.

To confirm that the direction of impact on intention was positive for both positive and negative normative messages, the study included a multivariate ANOVA including demographic variables and past physical activity behaviour (using IPAQ). To perform the ANOVA, for which the exogenous variables need to be categorical, IPAQ scores were transformed through a median split, resulting in two categories: the more active half vs. the less active half.

The distributions of intention presented a negative skewness (Positive Message Group skewness = −0.84 and kurtosis = 3.27; Negative Message Group skewness = −1.20 and kurtosis = 4.33; Control Group skewness = −0.79 and kurtosis = 3.26). To meet the assumptions of ANOVA, a positive transformation was used: −log(1 + maximum value of intention – intention)^(91/100). This transformation made the distribution of regression residuals normal (the null hypothesis of residuals being normal with a Skewness/Kurtosis test was accepted with p = 0.204) and therefore allowed a reliable interpretation of results.

**Figure 2:**
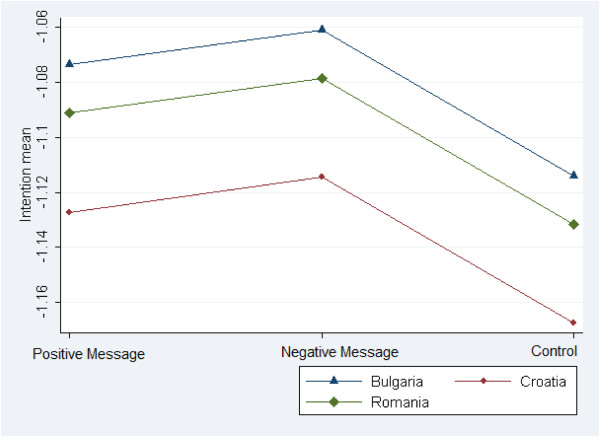
**Means of intention across control and treatment groups.**

Table 4 shows the results of the ANOVA model. This model compares the transformed intention means across groups. Significant differences were detected among the means of intention across the three treatments, the three countries and past physical activity behaviour. No interaction effect was significant, meaning that the normative messages had the same effect among these gender and country sub-populations. With regard to treatments and countries, a Tukey’s HSD test for pairwise comparison, studentized range critical value (0.05, 3, 1182) = 3.319, revealed that the only significant differences were that intention was (a) greater in the Positive Message Group than in the Control Group (mean difference = 0.046, HSD-test = 3.735), (b) greater in the Negative Message Group than in the Control Group (mean difference = 0.061, HSD-test = 4.940), (c) greater in Bulgaria than in Croatia (mean difference = 0.062, HSD-test = 5.001), and (d) greater in Romania than in Croatia (mean difference = 0.042, HSD-test = 3.413). Intention in the Negative Message Group was greater than in the Positive Message Group, although the difference was not statistically significant (mean difference = 0.015, HSD-test = 1.205). Figure 2 illustrates these results. With regard to past physical activity behaviour, a Tukey’s HSD test for pairwise comparison, studentized range critical value (0.05, 2, 1182) = 2.775, revealed significantly greater intention in the more active group than in the less active group (mean difference = 0.109, HSD-test = 10.775).

**Table 4:** **ANOVA model for intention to do physical activity**

Source	Partial SS	Df	F	Prob > F
Model***	5.434	17	5.25	0.000
Treatment*	0.484	2	3.97	0.019
**Gender**	0.165	1	2.72	0.100
Country**	0.694	2	5.70	0.003
**Age**	0.121	1	1.99	0.158
IPAQ***	3.501	1	57.49	0.000
**Interaction treatment/gender**	0.045	2	0.37	0.691
**Interaction treatment/country**	0.241	4	0.99	0.412
**Interaction treatment/age**	0.014	2	0.11	0.892
**Interaction treatment/IPAQ**	0.031	2	0.26	0.773
**Residual**	71.989	1182		
**Total**	77.423	1199		

Number of observations = 1200, Root MSE = 0.247, R-squared = 0.070, Adj R-squared = 0.057.

Significance levels: * = 0.05, ** = 0.01, *** = 0.001.

Finally, ANOVA showed a low fit (Adj-R^2^ = 0.057), meaning that although normative messages had a significant positive effect, they only accounted for a small amount of the total variation in intention to engage in physical activity.

## Discussion

Respondents exposed to a message suggesting most people of the same age, gender and country of residence as them were doing moderate physical activity (positive message) showed a significantly higher intention to be physically active than respondents who received no message. This result follows what was hypothesized and is in accordance with the principle of conformity to the norm and with previous research on the effect of descriptive norms on behaviour [17, 18, 22]. However, respondents presented with a message suggesting most people like them were not doing moderate physical activity (negative message) also showed a similar increase in intention to be physically active compared to respondents receiving no message. This result was unexpected. In fact, according to a second hypothesis, they should have demonstrated a lower intention compared to the control group, again following the principle of conformity to the norm.

It is possible that a *priming effect*, i.e. increased sensitivity to certain stimuli due to prior experience, accounts for this result [30]. People who are exposed to a message about the prevalence of physical activity among peers, including a photograph of people being active or being lazy, brings the issue of physical activity to the forefront of their attention. When asked, subsequently, to express their intention to undertake physical activity, they will have the information about peers' level of physical activity salient in their minds. This might lead them to undergo some self-monitoring task and make them more inclined to declare an intention to undertake physical activity in the near future, similar to the availability heuristic [30]. Previous studies which tested a normative message [17, 18, 22] did not control for a priming effect, and so this explanation cannot be ruled out.

The absence of a significant difference between the impact of the positive message and the negative message makes this last result even more unexpected. Assuming that a priming effect was taking place, the positive message should still have had a stronger effect on intention than the negative message because of the additive effects of conforming to the norm [17, 18, 31, 32] and priming [30]. On the other hand, the conformity to the norm effect of the negative message should operate in the opposite direction to the priming effect, resulting in a lower impact on intention than for the positive message. The study should be replicated with other samples, carefully separating a priming effect from a conformity-to-the-norm effect, to shed further light on the issue.

There are limitations to this research. For one, there was no measure of actual behaviour, only of intention to behave. Observing intention instead of behaviour is suboptimal from a health intervention point of view and limits the clinical significance of this study. Any public health attempt to change behaviour should not only seek to change intention, but also attempt to facilitate the translation of intentions into behaviour.

However, intention remains a useful construct. A number of behaviour change theories (most notably the theory of planned behaviour) consider intention a key factor in determining behaviour. Empirical studies support this claim. For instance, a meta-analysis examining the causal impact of intention on behaviour showed that a medium-to-large change in intention (*d* = 0.66) leads to a small-to-medium change in behaviour (*d* = 0.36) [33]. For physical activity, reviews and meta-analyses show that intention accounts for 20% to 40% of the explained change in behaviour [29, 34–36], and establish a moderate corrected meta-analytic correlation (r = 0.48) [37].

Finally, while the link between intention and behaviour is not straightforward, intention does capture the motivational forces that lead people to undertake physical activity over a period of time [38]. Observing changes in intention following different messages, therefore, can help identify which type of message is likely to be most effective in changing behaviour, after all other factors are taken into account.

More limitations apply. The results of this study are also based on the self-reported replies to an on-line questionnaire; therefore, all the limitations of self-reported data apply. The message that over 79% of people are physically active is rather high (normally the figure does not exceed 30%), and it may not have been considered believable by some participants. Also, there is a limitation on the external validity of the experiment, as the final sample of participants showed a difference in the drop-out rate between men and women. Finally, ANOVA's low explanatory power suggests that, although significant, the effect of the normative message is small. The objective of this study, however, was not to identify all the relevant factors affecting intention, rather to identify the effect on intention of positive and negative normative messages.

## Conclusion

Normative messages about the prevalence of peers' physical activity can increase intention to be physically active, regardless of whether the message claims the majority is doing or is *not* doing physical activity. For public health campaigns aiming to promote physical activity through normative messages, this is encouraging. The fact that these messages were individually tailored and engaged participants actively suggests that the Internet and mobile applications hold particular promise for such interventions. However, the fact that both positive and negative messaging elicited similar effects suggests other factors are involved, and invites further investigation.

## Endnote

^1^http://www.esomar.org/uploads/public/knowledge-and-standards/codes-and-guidelines/ESOMAR_Guideline-for-online-research.pdf
